# Hidden treasure of the Gobi: understanding how water limits range use of khulan in the Mongolian Gobi

**DOI:** 10.1038/s41598-020-59969-2

**Published:** 2020-02-19

**Authors:** John C. Payne, Bayarbaatar Buuveibaatar, Diana E. Bowler, Kirk A. Olson, Chris Walzer, Petra Kaczensky

**Affiliations:** 1 Research Institute of Wildlife Ecology, University of Veterinary Medicine Vienna, Vienna, Austria; 2 Wildlife Conservation Society (WCS), Ulaanbaatar, Mongolia & New York, USA; 3 Norwegian Institute of Nature Research (NINA), Trondheim, Norway; 4 German Centre for Integrative Biodiversity Research (iDiv) Halle-Jena-Leipzig, Leibzig, Germany; 5 Institute of Biodiversity, Friedrich Schiller University Jena, Jena, Germany; 6 UFZ – Helmholtz Centre for Environmental Research, Department of Ecosystem Services, Leipzig, Germany

**Keywords:** Animal migration, Conservation biology, Grassland ecology

## Abstract

Most large herbivores in arid landscapes need to drink which constrains their movements and makes them vulnerable to disturbance. Asiatic wild ass or khulan (*Equus hemionus*) were widespread and abundant throughout the arid landscapes of Central Asia and Mongolia, but have undergone dramatic population declines and range constrictions; denying khulan access to water is believed to have played a major role. Mongolia’s South Gobi Region now houses the world largest remaining khulan population, but is undergoing rapid land use changes. Khulan water use is poorly understood, largely due to the difficulty of mapping waterpoints used by khulan throughout their exceptionally large ranges, prone to high variations in precipitation. We used the special movement path characteristics of GPS tagged khulan to show us where water is located. We identified 367 waterpoints, 53 of which were of population importance, characterized the seasonal and circadian use, and identified snow cover as the most important variable predicting khulan visits during the non-growing season, and vegetation greenness during the growing season. Our results provide a data layer to help guide a regional khulan conservation strategy, allow predictions for other part of the global khulan range, and illustrates the overall importance of waterpoints for dryland herbivores.

## Introduction

Wildlife adapted to drylands have developed specific strategies to minimize water loss and optimize water recycling through anatomical, physiological, and behavioural adaptations^1,2^. Although some large herbivores can extract enough water from their food^3^, most need to drink regularly, and the availability of water strongly influences their daily, seasonal, or annual movements^4–7^. In arid environments standing water from precipitation is typically absent, thus requiring large herbivores to regularly access waterpoints. Because waterpoints tend to be scarce and spatially explicit, they constitute areas where herbivores are particularly vulnerable to competition, disturbance, and predation^8–10^. Hence, understanding the factors affecting water use in space and time is a precondition for understanding movement strategies and habitat use of large herbivores in arid ecosystems.

Water availability and access to water in the world’s drylands is increasingly driven by human exploitation of water for agriculture, industry, and domestic use. Livestock grazing is typically constrained to pastures close to water, thereby often reducing water and pasture accessible to wildlife^8,11–14^. Shared use of waterpoints harbours the risk of disease transmission between livestock and wildlife^15,16^. Finally, diversion of water for irrigation results in habitat conversion and when associated with fencing tends to block access to water by wildlife altogether. Actual or perceived competition with wild herbivores over water and nearby pastures often results in negative attitudes, facilitating retaliation actions including illegal killings particularly under drought conditions^17^. Identifying key waterpoints and understanding their temporal and seasonal use by water-dependent large herbivores is an important first step to guide conservation planning, including anti-poaching programs.

Revisits to waterpoints by wild and domestic herbivores result in movement patterns that are typically associated with central-place foragers, in which grazing is constrained by the need to return to a central resource^18,19^. Central-place foraging depletes forage available close to the central resource, resulting in wild and domestic herbivores selecting foraging areas away from water and encouraging them to switch between water sources^20^. Perpetuating such movement patterns requires high landscape permeability. Poorly located or unmitigated linear infrastructure such as fences, railways, or roads can block wildlife access to water and thereby reduce the area of pasture functionally available to wildlife. In turn, this can increase the dependence, and pressure, on the remaining accessible waterpoints^21–23^. Fragmentation of migration corridors to seasonally important water sources or reduced landscape permeability can result in mass mortality or population extinction^24^. Far-ranging, nomadic, and migratory ungulates are particularly sensitive to landscape fragmentation. Hence, identifying important waterpoints is essential for wildlife friendly land-use planning and the mitigation of infrastructure^25^.

Asiatic wild ass (*Equus hemionus;* regionally called khulan) once were widespread and abundant throughout the deserts and steppes of Central Asia and Mongolia. In modern times, they have become confined to less than 3% of their range, with a last stronghold in the Mongolian Gobi^26,27^. Population decreases and range contractions have been attributed to a combination of land conversion, overhunting, displacement by, and competition with livestock for pasture and water. In the former Central Asian Soviet Republics, where khulan went all-but-extinct by the 1940s, khulan may “have been ousted from most places without a shot fired. Water is scarce in the desert and steppes, and wherever man with his domestic animals settled by the rivers and springs, the wild animals were forced into waterless places and doomed to extinction”^28^.

Khulan, like all other equids, are hind gut fermenters, which enables them to process large quantities of low-quality food, but requires a large volume of water for microbial fermentation^29^. Khulan normally require 12–15 litres of water per day, and up to 24 litres on hot days. The low water content in their plant resources probably further increases their need to drink^28,30–32^. In the Mongolian Gobi, khulan roam over ranges of thousands of square kilometres and their movements are among the largest reported for terrestrial mammals^33–35^. Recent findings from the Dzungarian Gobi in south-western Mongolia suggest that water availability and switching among the sparsely located water bodies may be a key driver of the high mobility of khulan during the growing season^36^.

Droughts are common in the hot season of tropical or subtropical arid areas. However, in the cold winter deserts of Central Asia and Mongolia, lack of snow, the low water content of senescent vegetation, and the freezing of small and stagnant water bodies can also result in drought conditions during winter. In extreme cases these factors result in mass winter die-offs of livestock^37,38^. Khulan cope with localized catastrophic weather events by being highly mobile, but this requires habitat connectivity at a landscape scale^34,39–41^ and the protection of critical habitats that provide resources for population persistence^42^.

The Southern Gobi Region of Mongolia contains about 83% of khulan in Mongolia and 63% of the global population^26,43^. Large concentrations of khulan, numbering in the thousands, use specific waterpoints during certain seasons or years (C. Batsukh, Small Gobi Protected Area, pers. comm. 2014). Formal documentation of the location, visit intensity and seasonality of waterpoints used by khulan has been poor, in part due to the remote location of their range, the high intra- and interannual variation of rainfall^44^, and the difficulty to detect khulan diggings for water in dry riverbeds where subsurface flow exists on remote sensing products^45^.

Given the rapidity and scale of the changes in the Mongolian Gobi driven by market forces and the recent mining boom^46,47^, understanding the drivers and limitations of water use by khulan becomes ever more important as a basis for landscape-level conservation strategies. We addressed the current knowledge gap by analysing movement data from 41 khulan fitted with GPS tracking devices for periods up to 2.5 years between August 2013 and March 2018. To do so, we: (i) developed a custom-built algorithm to identify waterpoints from khulan trajectories, (ii) characterized khulan visitation of waterpoints in time and space, and (iii) evaluated the effect of climate conditions on the probability of visiting waterpoints. The results allow us to identify waterpoints which constitute a resource of population importance, estimate the average grazing area provided by an average waterpoint, assess the relative importance of climate driven factors on the probability of khulan visiting waterpoints, and provide a data layer to guide regional land use planning that is compatible with khulan conservation.

## Results

**Figure 1:**
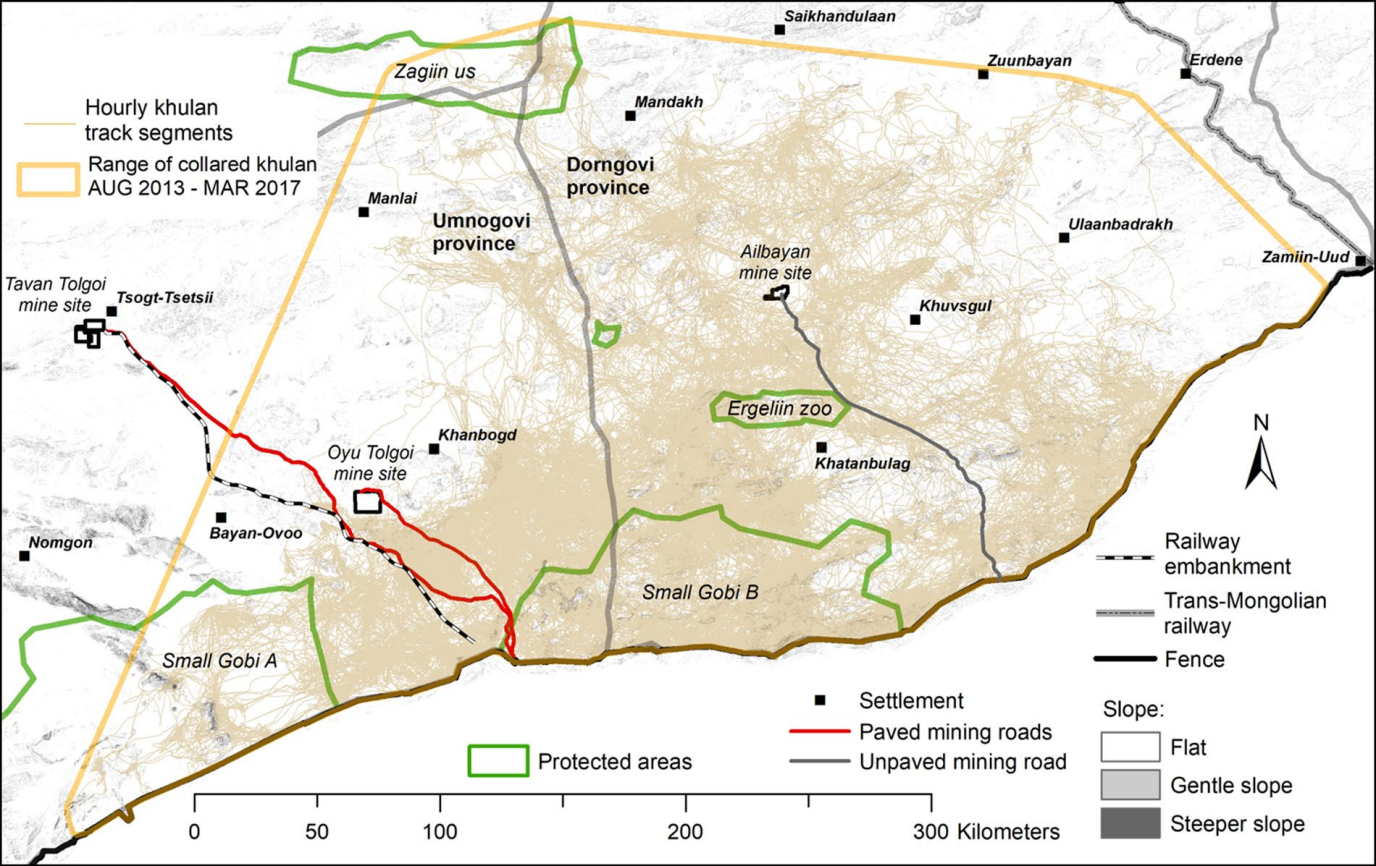
Map showing the GPS tracks (“trajectories”) of 41 khulan followed in the South Gobi Region between August 2013 until March 2018. The yellow polygon is the minimum convex polygon around all kulan locations (100% MCP) and delineates the area where waterpoints could be identified based on khulan trajectories. The southern border of the 100% MCP is defined by the fenced international border with China. Figure generated in ArcGIS 10.7.1 (ESRI, Redland, CA, USA, http://www.esri.com/).

Based on the trajectories of 41 GPS tagged khulan (Fig. 1), our water-finding algorithm identified 552 waterpoints based on annual khulan trajectories (Table 1), of which 367 represented unique (non-overlapping) waterpoint locations (Table 2). Ground-truthing confirmed the presence of water (either standing water, or evidence of recent water plus use by khulan) at all unique waterpoints that we opportunistically inspected during field work. Camera collar photographs and waterpoints mapped by colleagues provided additional proof and together verified 154 (42%) of the 367 unique waterpoints, lending high confidence in the assumption that waterpoints not verified are likely to be true waterpoints as well.

**Table 1:** The number of different waterpoints visited by individual khulan in the South Gobi Region.

Year	N waterpoints identified	N waterpoints visited per individual khulan each year				N months with active collars	Effort (collar-days)
		Median	Minimum	Maximum	Mean % of total		
2013	155	32	18	51	22	11	6,588
2014	136	25	7	34	17.9	12	5,377
2015	103	17	1	35	15.3	10	5,689
2016	107	24	10	40	23.8	12	5,261
2017**	51	5.5	2	18	14	9	1,610
**2013–2017**	**552**	**22**	**1**	**51**	**18.6**	**54**	**24,525**

^*^Year was defined from August 1 until July 31; **This year period ended after 8 months on March 31, 2018.

**Table 2:** Characteristics of 367 unique waterpoints grouped by intensity of use by khulan in the South Gobi Region from August 2013 to March 2018.

Waterpoint group	Total number		Median number of		
	Waterpoints	Visits	Visits	Visitors	Years
*Very high*	10 (2.7%)	2,464 (22.7%)	201.0	23.5	4
*High*	43 (11.7%)	3,615 (33.2%)	57.0	10	3
*Moderate*	48 (13.1%)	1,538 (14.1%)	29.5	6	2
*Low*	78 (21%)	1,500 (13.8%)	11.5	5	1
*Very low*	188 (51%)	1,766 (16.2%)	6.0	2	1
*Total*	367	10,883			

### Individual variation in the number of waterpoints visited

The median number of annual waterpoint visited in a given year by an individual khulan ranged between 17 and 32, excluding the truncated year, 2017. Individual khulan often returned to the same waterpoints repeatedly; 50% of consecutive visits were revisits to the same waterpoint. Nevertheless, the median number of different waterpoints visited by an individual khulan per year was 22 (Table 1).

### Intensity of waterpoint use

**Figure 2:**
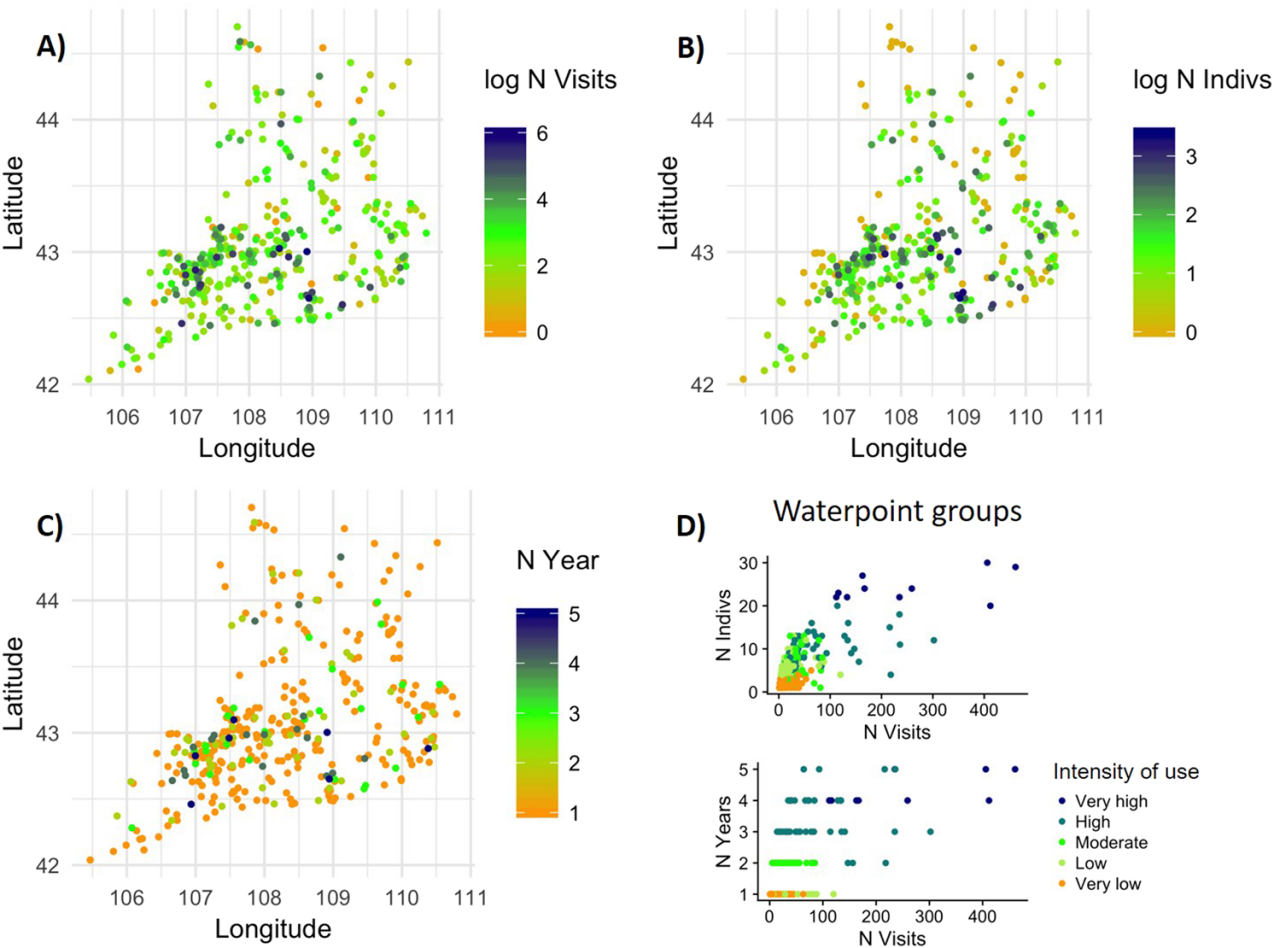
Location of unique waterpoints grouped by intensity of use by khulan in the South Gobi Region from August 2013 to March 2018 and pairwise relationships between use intensity variables. (**A**) Location by number of visits, (**B**) Location by number of visitors (different individual khulan), (**C**) Location by number of years visited. (**D**) Pair-wise scatterplots for the three variables defining intensity of use. For characterization of waterpoint categories please refer to Table 2.

Unique waterpoints were used very unevenly, with some receiving a much higher intensity of use than others. A cluster analysis based on the number of visits, number of visitors (different khulan), and years of use suggested 5 groups of waterpoints (Fig. S1 in SOM), with *Very high*, *High*, *Moderate*, *Low*, and *Very low* intensity of use. The *Very high* use group included 10 waterpoints which accounted for 23% of the total visits and had more than twice as many visitors as the *High* use group. Together, the two highest use groups (*Very high* and *High*) included only 14.4% of all unique waterpoints but received over half of the total visits (Table 2, Fig. 2).

At the low use end, there was a long tail of *Low-* or *Very Low* use waterpoints that were typically visited in only one year by 5 or fewer GPS tracked animals. However, these waterpoints collectively constituted 72% of all waterpoints and 30% of the total visits.

### Spatial and temporal patterns of waterpoint use

**Figure 3:**
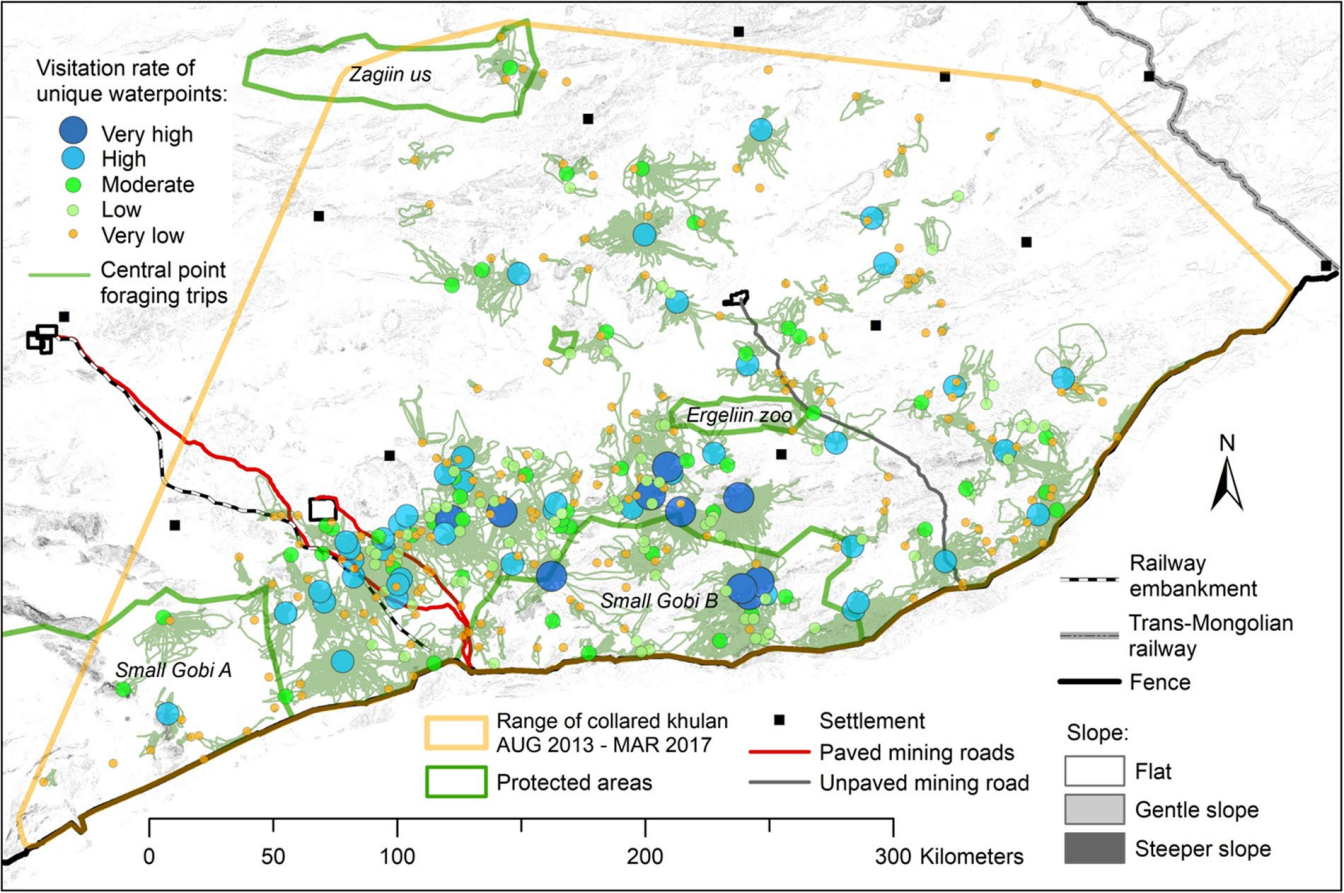
Location of 367 uniquewaterpoints grouped by the intensity of use and trajectories of revisits to the same waterpoint (*Central-place foraging trips*) by 41 khulan in the South Gobi Region from August 2013 and March 2018. For characterization of waterpoint categories please refer to Table 2. Figure generated in ArcGIS 10.7.1 (ESRI, Redland, CA, USA, http://www.esri.com/).

The unique waterpoints used by khulan were distributed throughout the khulan range but were particularly clustered along the edges of the Khanbogd massif south of Khanbogd, in the mining infrastructure corridor between the OT and TT roads, to the north of Small Gobi B SPA, and in the central-western part of Small Gobi B SPA (Figs. 3 and S2 in SOM). Only 64 of the 367 unique waterpoints (17.4% of waterpoints with 17.5% of all visits) were in protected areas, and only 11.3% of the waterpoints in the *Very high* (N = 4) and *High* (N = 2) use groups were in protected areas.

Khulan visited many of the frequently used unique waterpoints almost year-round, but visits were restricted to a smaller number of waterpoints in winter (N = 181) and spring (N = 203) as compared to summer (N = 237) and fall (N = 255). A total of 25% of all GPS locations were associated with *Central point foraging trips*, i.e. consecutive revisits to the same waterpoint by an individual khulan, another 15% with *Inter-waterpoint transitions*, i.e. switching between track-based waterpoints, and the remaining 60% of the GPS locations were associated with *Other* tracks.

*Central point foraging* trips had a median track length of 17.3 km (mean: 20.5 km, range: 0.3–132.2 km), a median duration of 26 hours (mean: 30 hours, range: 5–72 hours (our cut-off time)) and brought the khulan a median distance of 7.2 km (mean: 8.6 km, range: 0.7–58.0 km) away from the waterpoint at the furthest point. Using the median distance of the farthest track point from the waterpoint as the radius of a circle, the potential grazing range for khulan around an average waterpoint is 163 km^2^.

**Figure 4:**
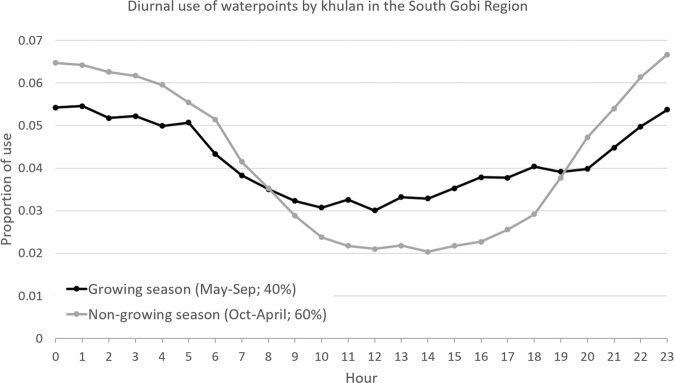
Diurnal pattern of khulan visits to waterpoints in the South Gobi Region. Of the 33,019 hourly positions at waterpoints, 40% fell in the 5-month growing season and 60% in the 7-month non-growing season.

Khulan visited waterpoints most often around midnight and were least likely to go to water between late morning and early afternoon, particularly in winter (Fig. 4). The median duration of a khulan to remain at a waterpoint was 2 hours.

### Impact of environmental variables on drinking frequency

On average, individual khulan visited a waterpoint every 1–2 days (mean: 2.1 days, median: 1.0 days, SD: 5.2, range: 0.04–109.2 days). Individual variation in the drinking frequency was high, with some individuals going for long periods without visiting any known waterpoint, likely relying on small unmapped water sources.

**Figure 5:**
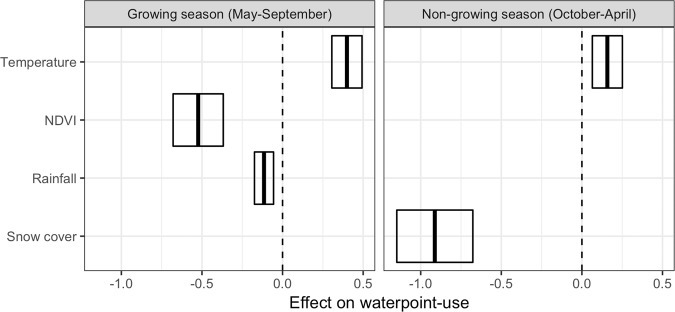
Effect sizes of each climate variable on the probability of khulan visiting a waterpoint. Boxes represent the means and 95% confidence intervals of the standardized effect sizes (change in the probability of waterpoint use on a logit scale per standard deviation change in each variable). If a box does not overlap with the dashed no-effect line, the influence of the variable is significant at 0.05.

Daily variation in the probability of a khulan visiting a waterpoint was clearly influenced by climate, despite individual variability (Fig. S4 in SOM). The model for the growing season showed a strong negative effect of NDVI on the probability of visiting a waterpoint, a small additional negative effect of rainfall and a moderate positive effect of average temperature (Fig. 5). The model for the non-growing season showed a very strong negative effect of snow cover (twice as strong as the effect of NDVI in the summer model) and a small additional positive effect of temperature (Fig. 5). Dispersion parameters (ratio of residual deviance to residual degrees of freedom) were close to 1 for both seasonal models, suggesting goodness-of-fit, and the adjusted R^2^ was 18.2% for the non-growing and 14.6% for the growing season model.

## Discussion

### Using a water-dependent large herbivore to map waterpoints

Mapping water availability in the Gobi and other non-equilibrium drylands is challenging as many water bodies are only intermittently available, or so small in dimension, that they are not detected by automated water classification tools^48^. However, the specific water-seeking movements of khulan allowed us to use their GPS trajectories to identify waterpoints, most of which would not have been visible on remote sensing products. The simple algorithm we developed proved robust, as confirmed by the evidence of water at a subset of the predicted locations visited during opportunistic ground-truthing, the absence of false negatives, and the ability to repeatedly identify waterpoints that were used years apart by animals not traveling together.

The number of waterpoints identified by our algorithm tends to increase with GPS tracking effort, in part due to the high variability in water availability (i.e. ephemeral waterbodies, which are not spatially fixed, accumulate in area over time) and individual differences (i.e. khulan that went for long periods without visiting any known waterpoints obviously accessed water somewhere). Analyzing waterpoints by year and pooling over all individuals helped to reduce this bias and made it possible to distinguish between waterpoints that were used consistently over multiple years (likely permanent waterpoints) and temporary or ephemeral sources. By its nature, the algorithm can only detect waterpoints used by khulan, not all waterpoints available, and therefore provides only limited insight into khulan choice. However, the latter was not the focus of our work.

The trajectories of Savannah elephants (*Loxodonta africana*) going to water appear to be very similar to those of khulan^6^. Other highly mobile and water dependent large herbivores living in drylands face similar constraints and are likely to also approach water in a very directed way, so that our algorithm may be applicable in other regions where waterpoints are difficult to map. Detecting waterpoints with the help of one species with specific water seeking-movements can also help understand movements of other species; for example, we found that collared goitered gazelles used many of the khulan waterpoints in the areas where they overlapped (BB unpubl. data).

### Waterpoints constrain khulan movements

We identified a set of 10 *Very highly* frequented and 43 *Highly* frequented key waterpoints, which were consistently used by many different khulan over multiple years. This consistent use is remarkable as all khulan travelled independently of each other, were collared during two different capture events spaced two years apart, and in two different capture locations spaced >150 km apart. The combined range of the 20 khulan captured in 2013 was almost identical with the range of those captured in 2015 and matched the range from independent surveys from 2012–2015^27,43^. We are therefore confident, that our 41 collared khulan constitute a representative sample of the larger khulan population in the South Gobi Region and that the 53 most frequented waterpoints constitute a resource of population-level importance.

Khulan visited known waterpoints every 1–2 days and about half of all visits were returns to the same waterpoint, with individual khulan using ca. 20 different waterpoints annually. This puts khulan into the group of poorly-studied central-place foraging herbivores which are constrained in their foraging movements by the need to return to a central resource, i.e. water for khulan^18^. This need to return to a central place restricts the pasture potentially available for grazing to species-specific commuting distances^8^ and results in pasture depletion close to the central source^20^. Switching between resources allows large herbivores more flexibility to avoid competition close to the central resource (with other khulan or other wild and domestic ungulates). But not only access to the key waterpoints is important; the smaller, ephemeral, or less preferred waterpoints act as stepping stones to enable khulan to switch between distant parts of a khulan’s range (Fig. S5) or to access pastures where competition, disturbance, or predation is lower^36^.

### Variables influencing khulan drinking frequency

The visitation frequency of waterpoints by khulan was clearly influenced by climate-driven factors. During the growing season, khulan were less likely to visit waterpoints when the vegetation was green, but more likely to visit waterpoints when the temperature was high or when it had not recently rained. During the non-growing season, snow cover greatly reduced the probability of khulan visiting waterpoints, to the point that when most of the range was covered by snow, khulan almost completely stopped going to water; mean daily temperature during the non-growing season had only a small positive effect on visitation probability. Our model provides the first quantitative assessment of the different climate variables allowing us to predict water dependence of the species in time and space throughout its global range and under different climate and disturbance scenarios.

Snow cover was the most important variable predicting waterpoint use during the non-growing season. In the Dzungarian Gobi in south-western Mongolia, where winter snow is a constant, it reliably liberates khulan from the need to visit waterpoints in winter^31^ (Fig. S6 in SOM). This provides khulan access to additional pastures in winter, thereby reducing grazing pressure during a critical time of the year. However, when snow is absent and very cold temperatures result in small or stagnant waterbodies freezing solidly, the availability of waterpoints with flowing water may be even more reduced than during a summer drought.

Greenness of the vegetation, assessed via NDVI, was the most important factor during the growing season predicting the probability that a khulan would visit a waterpoint. Green-up and greenness of the vegetation in drylands is tightly linked to precipitation and reflects the water content of the plant (which is tied to photosynthetic activity)^49^. Whereas intense rain events provide immediate, but very short-term access to drinking water in the form of rain puddles throughout the impact area, the rainwater absorbed by the vegetation becomes available to khulan over longer periods in the form of green forage. Hence, the longer-term NDVI produced a stronger signal than the immediate rain, which had a relatively small additional effect.

Ambient summer temperatures in the South Gobi Region easily reach 30 °C and above. Domestic equids have a thermal neutral zone somewhere in the range of 5–30 °C when standing still^50,51^ and khulan can be expected to have a similar range. At higher temperatures or during exercise, khulan need to evaporate water to cool themselves down, which explains why higher temperatures were associated with an increased probability of khulan visiting a waterpoint.

Under a warming climate (higher evaporation, increased heat stress of plants) or an increased disturbance regime, the need to access water can be expected to increase and may reduce the radius at which khulan can graze away from water, thus reducing the pasture area that is functionally available for the khulan population. Furthermore, the frequency of extreme events like droughts and extreme winters (both with no snow or extreme amounts of snow) is expected to further increase^52,53^. The high mobility of khulan and other migratory wild ungulates is the best strategy to cope with this uncertainty, but requires a high level of landscape connectivity^39,54^.

### Conservation implications

Our 100,000 km^2^ study area constitutes the core of the world largest remaining khulan population^27^. We identified a set of 53 waterpoints which must be considered as having population-level importance (key waterpoints). At the same time, there is clear evidence that khulan are negatively impacted by human activities and livestock presence^34,43,55,56^. To ensure the long-term survival of the khulan in the South Gobi Region, key waterpoints should receive some form of protection that at a minimum ensures unlimited access by wildlife and limits disturbance to the surrounding habitat. Currently, only 6 of these most-frequented waterpoints are located within national protected areas. This is a matter of concern, especially considering the rapidly increasing livestock numbers (in our study, livestock numbers have increased by almost 40% within our 5-year study period (Fig. S7 in SOM) and the ongoing infrastructure developments in the South Gobi Region^46^.

Khulan seem to respond to the risk of disturbance at waterpoints, which includes illegal killings^57,58^, by visiting these locations primarily at night. In the Western Gobi Region, where humans and livestock are absent during most of the growing season, khulan visit waterpoints almost equally over all 24 hours of the day (Fig. S6 in SOM). Temporal niche sharing, allowing the coexistence of wildlife and humans in multi-use landscapes has been widely documented elsewhere^59^. In the khulan conservation context, it requires humans to remain at a certain distance from waterpoints, especially at night so that khulan and other wildlife can access waterpoints without being disturbed by unpredictable human activity such as off-road vehicles or free-ranging dogs^60^. Furthermore, anti-poaching units should regular patrol key waterpoints to discourage poaching. Given the fundamental importance of waterpoints in the Gobi for wildlife, livestock, and people, a respective “code of conduct” should be drafted together with local stakeholders. Such a document should incorporate existing informal rules which regulate minimum distances for camps, water use, and foresee some form of monitoring of water quality and flow. Using citizen science to monitor wildlife and water at key waterpoints could help to establish ownership, pride, and raise awareness for the unique Gobi fauna and water as a precious resource, as has been demonstrated for other environmental contexts elsewhere^61^.

Several of the waterpoints highly frequented by khulan are near mining infrastructure, which demonstrates that khulan will habituate to the infrastructure (including traffic which predictably stays on the road) to gain access to water. Nevertheless, there is strong evidence that even roads with moderate traffic constitute considerable movement barriers (e.g. crossings of the Oyu Tolgoi mining road were about 50% less than expected and crossings occurred primarily at night when traffic volumes are minimal; JP, BB, PK unpubl. data). Current traffic volumes on mining roads are low to moderate and largely confined to daylight hours. However, with additional development and economic opportunities, traffic will likely increase, as will be the need for additional transportation corridors, hence resulting in stronger barrier effects^62,63^. To avoid cutting off khulan from waterpoints of population-level importance, these locations should be flagged as *no-go* zones for new infrastructure and development projects, and existing infrastructure should be realigned where possible or else its impact mitigated (e.g. via wildlife underpasses, implemented based on the requirements and standards for mitigating transportation corridors in Mongolia from 2015).

However, the large ranges covered by individual khulan and the large number of waterpoints visited by the population of GPS-collared khulan highlights once again the need to ensure that landscape connectivity is maintained. The non-overlapping potential grazing range of the 53 key waterpoints sums up to a maximum of 7,208 km^2^, which is at most one quarter the size of the area that a single khulan covers within 1 or 2 years. Experience from other regions has shown that variable, low productivity landscapes will only support a small fraction of the number of ungulates if migration is no longer possible^64^. Hence, just protecting key waterpoints and their surrounding pastures is clearly not enough.

Only by giving khulan and other nomadic, far-ranging or migratory ungulates the flexibility to access a large number of waterpoints to react to changes in pasture and water availability will it be possible to safeguard the unique migratory landscapes of Mongolia and Central Asia (Convention of Migratory Mammals Central Asian Mammals Initiative at https://www.cms.int/node/28). Hence, avoiding dramatic population declines will require a regional conservation strategy that identifies areas that should be formally protected, while at the same time promoting and protecting the high degree of connectivity in the multi-use landscape that currently exists.

## Material and Methods

### Study area

The study area encompassed ca. 100,000 km^2^, dominated by arid plains, interspersed with hills and low mountain ranges with elevations of 683–1,884 m (Fig. 1). Five national level protected areas intersect the study area: Small Gobi A and Small Gobi B Strictly Protected Areas (totalling 18,000 km^2^), Ergeliin Zoo Nature Reserve (NR; 605 km^2^), Zagiin Us NR (2,740 km^2^), and Suikhent NR (55 km^2^). The southern and eastern border of the khulan range is defined by two absolute barriers; the fenced international border between Mongolia and China and the fenced Trans-Mongolian railway^23^. The northern border is likely defined by high livestock densities^34^. What limits the range along the western edge is not fully understood but may include heavy poaching in the past^65^.

**Figure 6:**
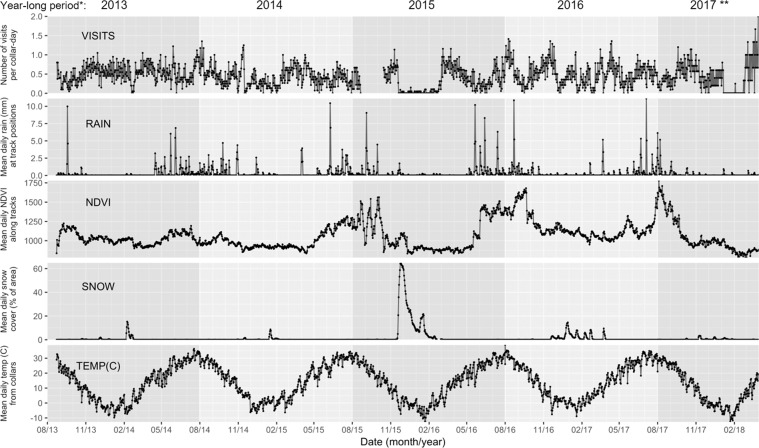
Mean daily visitation rates by khulan to waterpoints and matching environmental variables in the South Gobi Region August 2013 to March 2018. *Year-long periods were defined from August 1–July 31 and are shown with alternating background shading. **The 2017 period ended after 8 months on March 31, 2018.

The average annual temperature is around 7 °C, but daily extremes vary between 40 °C in summer and −30 °C in winter. Average annual precipitation increases from the southwest to the northeast from an average 100 mm to around 150 mm. Precipitation is highly variable within and between years, but usually peaks during summer (June to August). In winter, additional precipitation can occur in the form of snow, but only irregularly (Fig. 6).

Surface water is available to wildlife at springs, many of which are only temporary, and ephemeral pools in natural depressions fed by snow melt or rainwater. Khulan are also capable of accessing water by digging in dry riverbeds, in places where there is subsurface flow (i.e. the water table is close to the surface^45^). These diggings tend to be less than 0.5 m across, up to 0.5 m deep, at times can be totally covered by sand, are not associated with any vegetation (Fig. S8 in SOM) and hence are not visible on open source remote sensing products^48,66,67^. Although the availability of water at springs, pools, or diggings in dry riverbeds varies in time, their location is more or less spatially explicit; in other words, *when* water is available is more variable than *where* it is available.

Vegetation is sparse and dominated by desert-steppe and semi-desert plant communities, particularly *Artemisia* spp., *Allium* spp., *Stipa* spp., and *Anabasis brevifolia*. There are a few tree species, including saxaul (*Haloxylon ammodendron*), elm (*Ulmus pumila*), and poplar (*Populus diversifolia*), which are confined to river valleys and large basins^68^.

The khulan population in the Southern Gobi Region of Mongolia is estimated to number approximately 35,000 individuals^27^. Other large mammalian wildlife in the area include goitered gazelles (*Gazella subgutturosa*) and Mongolian gazelle (*Procapra gutturosa*) on the plains, and argali wild sheep (*Ovis ammon*) and ibex (*Capra sibirica*) in the mountains. Large mammalian carnivores include grey wolf (*Canis lupus*), Eurasian lynx (*Lynx lynx*), and snow leopard (*Panthera uncia*).

The region is at the center of the cashmere goat industry in Mongolia, and livestock products generate the main income of local herders^47^. The rural populations consist of semi-nomadic livestock herders, in 2018 amounting to 3,281 herding households with 1.2 million head of livestock (53% goats, 32% sheep, 6.5% camels, 5.7% horses, and 2.7% cattle) in those 7 districts which intersect the study area (Mongolian Statistical Information Service 2019 at http://www.1212.mn/en/; Fig. S7 in SOM).

Recent, rapid development of the mining sector has resulted in new mining related infrastructure and an influx of people, especially around the Oyu Tolgoi copper and gold mine near Khanbogd and the Tavan Tolgoi coal mine near Tsogttsetsii, and also at export hubs to neighbouring China, including Zamiin-Uud border crossing, which is the exit and entry-point of the Trans-Mongolian railway to and from Beijing. Three regularly frequented mining roads are currently found within the core of the khulan population: the paved Oyu Tolgoi road (32 vehicles/hour in 2016) and Tavan Tolgoi road (44 vehicles/hour in 2016) connecting the mine sites to the Gashuun Sukhait border crossing and the unpaved Ailbayan road (2.5 vehicles/hour in 2016) connecting the Ailbayan coal mine to the Khangi border crossing. Other Gobi roads in 2016 had traffic volumes of <1 vehicle/hour (BB, JP, PK, unpubl. Data). Further roads and railways are in the process of being built (e.g. the embankment for a railway parallel to the Tavan Tolgoi road is already in place), approved, or planned^25^.

### Methods

#### Animal capture and monitoring

We anesthetized and collared a total of 41 khulan (21 males and 20 females) with GPS satellite collars (Vertex Plus 4D from Vectronic Aerospace, Germany & IridiumTrackM 3D from Wireless Inc., Canada) during two capture events in August 2013 and October 2015. Capture and anesthesia followed methods previously described^69–71^. All captures and animal handling were performed in accordance with relevant guidelines and regulations. The capture was authorized by the Mongolian Ministry of Nature, Environment and Tourism (capture permits 6/4136 issued 2013/08/01 and 5/5656 issued 2015/09/17) and the ethic commission at the University of Veterinary Medicine Vienna was informed and provided general consent (ETK-15/03/2016).

The collars we deployed on khulan were programmed to record GPS locations at hourly intervals, and to last for 2.0–2.5 years. All collars were equipped with pre-programmed drop-offs (CR-2A, Telonics, USA) for animal welfare reasons and to allow collar retrieval. GPS locations were loaded into a PostgreSQL database and quality-controlled. This resulted in a final set of 589,708 hourly GPS locations with monitoring periods of individual khulan lasting 53–827 days (median = 700 days). The individual areas the collared khulan covered during the monitoring period were large, ranging between 10,971–63,606 km^2^ (median: 31,804 km^2^, expressed as 100% Minimum Convex Polygons (MCPs); Table S1 in SOM).

#### Identifying water points

Existing tools for analysing tracking data are inadequate for identifying waterpoints in the South Gobi Region, because they rely on some combination of point density, movement recursion, or residence time (see for example the “adehabitatHR” library in R^72^). Khulan spend very little time at a waterpoint and frequently switch between different waterpoints, which limits the usefulness of measurements based on point density or residence times. Measures of recursion are potentially more useful, but initial tests showed they also identify other locations that are revisited, including corridors or other areas of attraction (data not shown).

We noted that khulan tracks tended to be characterized by long, directed movements when approaching water, a small number (often only one or two) of GPS locations in the vicinity of the water source, followed by an equally long and directed movement away from water back in the direction the animal had come from. This pattern became particularly obvious in the snowless winter of 2013/14, when it resulted in the massive convergence of khulan trajectories to a number of locations which upon ground inspection in March 2014 were all confirmed to be waterpoints (PK, JP, BB unpubl. Data; also see Fig. S5 in SOM).

Based on these observations, we designed a two-step water-finding algorithm that in a first step identified points where individual khulan turned around sharply (“turnpoints”) and in a second step discarded as false-positives those turnpoints that were not clustered together. Buffers drawn around the remaining clustered turnpoints were joined into shapes that we interpreted as a probabilistic representation of waterpoint locations (Fig. S9 in SOM; parameters are further explained below and values for *t*, *d*, *L β*, and *α* are given at the end of this paragraph).

1. To locate turnpoints, the algorithm moved a 7-hour window along each khulan track of hourly GPS locations. At each position, it drew a line between the central position in the 7-hour window and the GPS locations at the beginning and the end of the window (“3-hour approach vector” and “3-hour departure vector”, respectively) and between the central position and the GPS locations one hour earlier and later (“1-hour approach and departure vectors”, respectively). The algorithm identified as turnpoints only those track points for which the 3-hour approach and departure vectors were both longer than length *L* and which were separated by a turn angle no larger than *β*. An additional condition, that the turn angle between the 1-hour approach and departure vectors must be less than *α*, was included to make the algorithm slightly more selective and to better identify the point at which the direction had changed.
2. To define waterpoints, first the turnpoints from all animals in a given year were collected. Next, a circular buffer of diameter *d* was drawn around each turnpoint. If there were fewer than *t* turnpoints in a buffer, the focal turnpoint was assumed to be false-positive and was dropped. The remaining buffers were merged into shapes that we subsequently referred to as annual waterpoints (Fig. S10 in SOM).

The number of turnpoints identified by the algorithm is correlated with the total number of hourly GPS positions. To address this bias, we ran the algorithm separately for year-long periods broken on August 1^st^ to best match the general timing of collar deployments (Table S1 in SOM) and to align with refilling of aquifers following summer rains (Fig. 6). Note that the last period, 2017, was only 8 months long due to the end of monitoring on 31 March 2018.

To calibrate the model for the South Gobi Region, we analyzed khulan tracking data from the much smaller Great Gobi B Strictly Protected Area (SPA; 9,000 km^2^) in the western Gobi, where the waterpoints are largely permanent and well mapped^36,73^. The goal of the calibration was to find a balance between not missing known waterpoints and generating the fewest possible false-positives. We examined a range of lengths *L* for the approach and departure vectors from 1 to 4 km, and for the angles *α* and *β* from 45 to 120 degrees, for the buffer diameter *d* from 300 to 700 m, and threshold for dropping points *t* from 2 to 3 (Data not shown). Several related parameter sets performed well, with the best parameter set being: *L* ≥ 2 km, *α* = *β* < 90°, *d* = 700 m, and *t* ≥ 2 (i.e., single turnpoints that did not have at least one other turnpoint within their buffer were discarded). Using the above criteria, the number of turnpoints used to identified waterpoints represented 0.81% of all track positions.

#### Ground-truthing waterpoints

We opportunistically ground-truthed waterpoints for the presence of water or signs of recent khulan use (direct observations, tracks, dung) through: (1) field visits in March 2014 to inspect locations where many khulan tracks converged in the snowless winter of 2013/14, (2) field visits in July 2017 to waterpoints identified by the water finding algorithm that fell near our travel route, (3) photographs of waterpoints taken by a khulan with a camera collar at locations that overlapped with waterpoints identified by our water-finding algorithm^56^, and 4) a list of 45 waterpoints mapped by other researchers during field work 2013–2017 that overlapped with waterpoints identified by our water-finding algorithm.

#### Analysing waterpoint visitation patterns

Depending on the analysis question, we either 1) considered annual waterpoints for time specific analysis from the khulan’s perspective (e.g. visits in a particular year by individuals) or 2) derived unique waterpoints by merging those annual waterpoints which overlapped spatially for analysis from the waterpoint’s perspective (e.g. number of visits by khulans, number of years visited). For displaying the locations of unique track-based waterpoints, we used the coordinates of the centroid of the intersection of the annual waterpoint shapes (i.e., the center of the area of highest overlap).

The intersection of a khulan’s GPS location with the shape of an annual waterpoint was recorded as a visit to the waterpoint. Each visit was identified with a visit ID number. To avoid double-counting visits when a khulan lingered by a waterpoint, we gave the same visit ID number to sequential track locations that occurred at the same waterpoint, unless the sequence was interrupted by either a break of more than 5 hours or a visit to a different waterpoint. Various statistics were calculated based on these visit IDs, from both the khulan perspective (number of different waterpoints visited per year, number of visits per day, and time of visits) and the waterpoint perspective (number of visits, number of visitors, number of years visited, and season of the visit). For seasonal analysis in respect to conservation planning (e.g. is there a particular season a highly-frequented waterpoint is used most, making it more important to limit livestock access or have ranger presence in the area), we used the 3-month periods most commonly used for seasons in Mongolia, with March-May for spring, June-August for summer, September-November for fall, and December-February for winter.

To identify unique waterpoints with similar patterns of use, we applied a hierarchical cluster analysis (Ward algorithm^74^) to data on waterpoint use (the number of total visits, the number of unique individuals of the visits, and the number of years the waterpoint was visited). We plotted the relationship between the number of clusters and the within-cluster variation to assess the appropriate number of clusters (Fig. S1 in SOM).

We explored overall foraging behavior by categorizing all GPS positions as falling into 3 categories:

1. *Central point foraging trips*: track positions that fell between consecutive visits to the same waterpoint, where the duration of the trip was no more than 3 days;
2. *Inter-waterpoint transitions*: track positions that fell between consecutive visits to waterpoints where the start and endpoints were at different waterpoints; again, the duration of the trip was no more than 3 days;
3. Other track positions.

To characterize *Central point foraging trips*, we drew minimum convex polygons around the GPS locations comprising foraging trips to calculate: track length, maximum distance away from a waterpoint, and duration between consecutive visits. We additionally calculated the median size of the grazing area made available by an average waterpoint, using the area of a circle where the radius was the median of the maximum distance away from a waterpoint.

#### The influence of environmental variables on waterpoint use and visitation rates

Environmental variables: To test which environmental variables influence waterpoint visitation rates, we obtained values for precipitation, snow cover, vegetation greenness (as a proxy for plant water content), and ambient temperature (Fig. 6) from the following sources:

1. Precipitation from the Global Precipitation Climatology Centre’s GPCC First Guess Daily product^75^ available on a 1-degree grid (roughly 110 km longitude × 82 km latitude, at the latitude of the study area) and available open source at: https://opendata.dwd.de/climate_environment/GPCC/html/gpcc_firstguess_daily_doi_download.html. We had previously found good agreement of this landscape-scale precipitation data with localized rain information from a weather station at the Oyu Tolgoi mine site (JP unpubl. data) and evidence of rain from camera collar images^56^. For each khulan, we calculated the daily rainfall as the mean of the grid values which were intersected by its hourly GPS positions.
2. Vegetation greenness based on the Normalized Vegetation Index (NDVI) from the 250 m Grid, 16-day composite MODIS/Terra Vegetation Indices product (Version 6, MOD13Q1, open source available from the SGS website at: https://e4ftl01.cr.usgs.gov/MOLT/MOD13Q1.006/. We mosaiced and re-projected the tiles (from *Sin* to *Geographic*), extracted the NDVI index and NASA’s pixel reliability layer^76^. We simplified the pixel reliability layer by converting all categories except “*Good data*” to missing-data values, then used the resulting layer as a mask to exclude questionable pixels from the NDVI data. We subsequently loaded the layers into PostgreSQL database tables, using a custom R function and NASA’s *MODIS Reprojection Tool* available at: https://lpdaac.usgs.gov/tools/modis_reprojection_tool. For each khulan we calculated the daily mean NDVI based on 9 pixels around each hourly GPS position.
3. Snow cover from the MODIS/Terra Snow Cover Daily L3 Global 500 m Grid, Version 6 (MOD10A1; available at: http://nsidc.org/data/mod10a1). Satellite images of the Gobi in winter are often obscured by cloud, sometimes over very large areas for days on end. Matching khulan track locations with individual pixels from the daily snow cover images resulted in time series with many missing values. We therefore calculated a 5-day running mean of the proportion of snow cover for the entire khulan range (100% minimum convex polygon of all track positions, Fig. 1) for each day, and the same daily snow cover values were assigned to each animal.
4. Ambient temperature from the hourly temperatures measured by sensors in each khulan collar (as a rough proxy for the temperatures experienced by the animals). For each khulan we calculated the daily mean of the collar temperature and assigned individual daily means to each khulan.

GAM modeling: To test the effect of abiotic variables on water use, we modelled the probability of a waterpoint visit by a khulan on a given day as a binary variable in a generalized additive mixed effects model^77^. We tested the effects of climate variables snow cover, temperature, rainfall and NDVI as fixed effects in two separate models because snow and rain/NDVI are more or less mutually exclusive. For the growing season (May to September) we tested for the effect of temperature, rainfall and NDVI and for the non-growing season (October–April) for the effect of temperature and snow cover. These variables were standardized to units of standard deviations so that their effect sizes represented their relative importance. We included a random intercept for individual ID and random slopes for each individual for the effect of each abiotic variable. To account for spatial autocorrelation, we also included a Duchon spline term for the interaction of latitude and longitude of the centroid of each individual’s daily movement track (Fig. S2 in SOM). Because the response was a binary variable, we specified the family argument as binomial. These models were fitted with the R package *gamm4*^77^ (R script provided in the SOM).

## Supplementary information

Supplementary Information.

## Data Availability

The raw data on waterpoint characterizations and water visitation organized by individual khulan and day can be accessed from Dryad Digital Repository 10.5061/dryad.pg4f4qrjw.
