# Supplementary material for: Hidden treasure of the Gobi: understanding how water limits range use of khulan in the Mongolian Gobi
Source: Sci Rep. 2020 Feb 19;10:2989. doi: 10.1038/s41598-020-59969-2 (PMC7031417; doi:10.1038/s41598-020-59969-2)
Supplement: Supplementary file 1 — Supplementary Information. [file 41598_2020_59969_MOESM1_ESM.pdf]

Supplementary information for:

Hidden treasure of the Gobi: understanding how water limits range use of khulan in the Mongolian Gobi

John Payne[1,2], Bayarbaatar Buuveibaatar[2], Diana Bowler[3,4], Kirk Olson[2], Chris Walzer[1,2] Petra Kaczensky[2,1*]

*Corresponding author: petra.kaczensky@nina.no

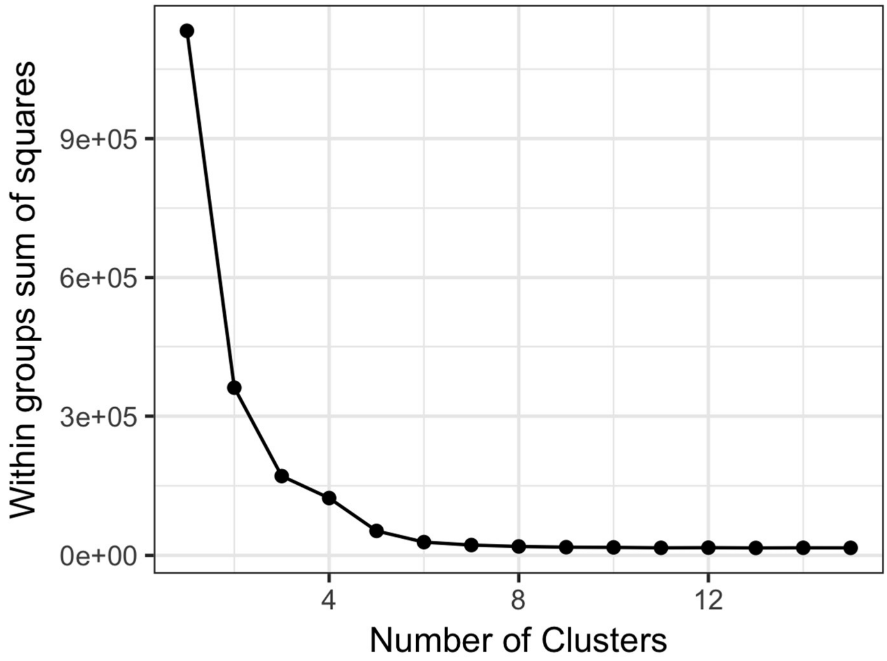

Fig. S1: Relationship between the number of clusters and the within-cluster variation, to assess the appropriate number of clusters for categorization of unique waterpoints based on number of visits, number of visitors, and years visited.

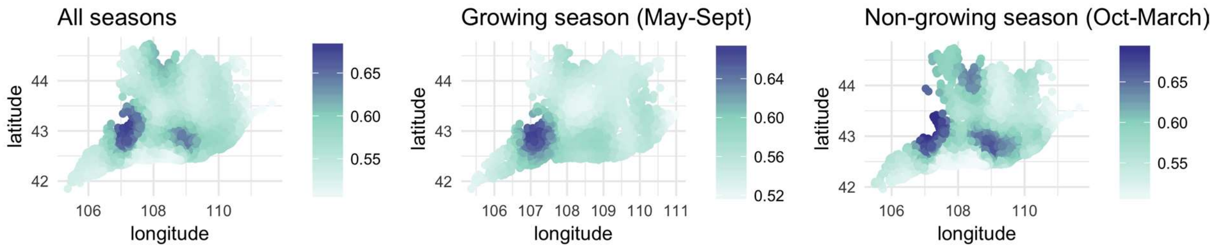

Fig. S2: Spatial variation in the probability of khulan visiting waterpoints in the SE Gobi 2013-2017. The spatial surface pattern is based on the XY coordinates of the centroids of the daily positions of each animal and was included in the GAM.

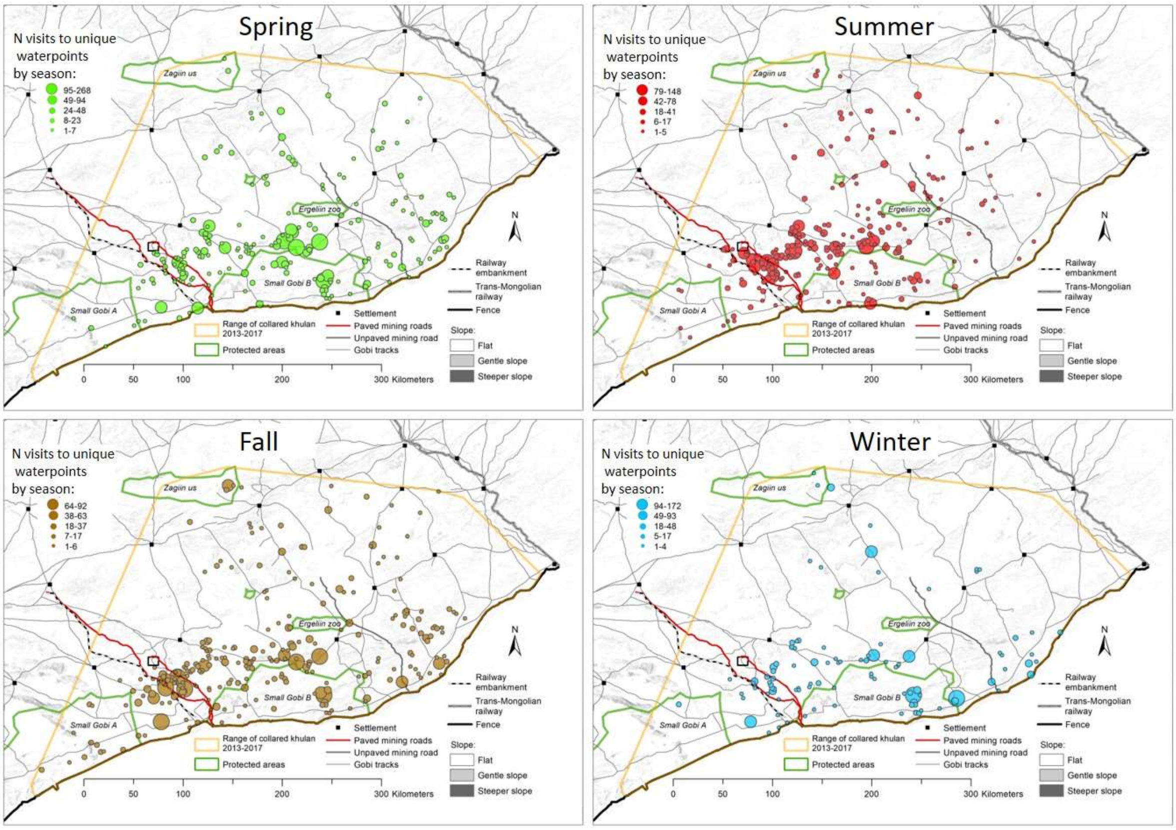

Fig. S3: Seasonal use of unique waterpoints by khulan in the South Gobi Region from August 2013 to March 2018.

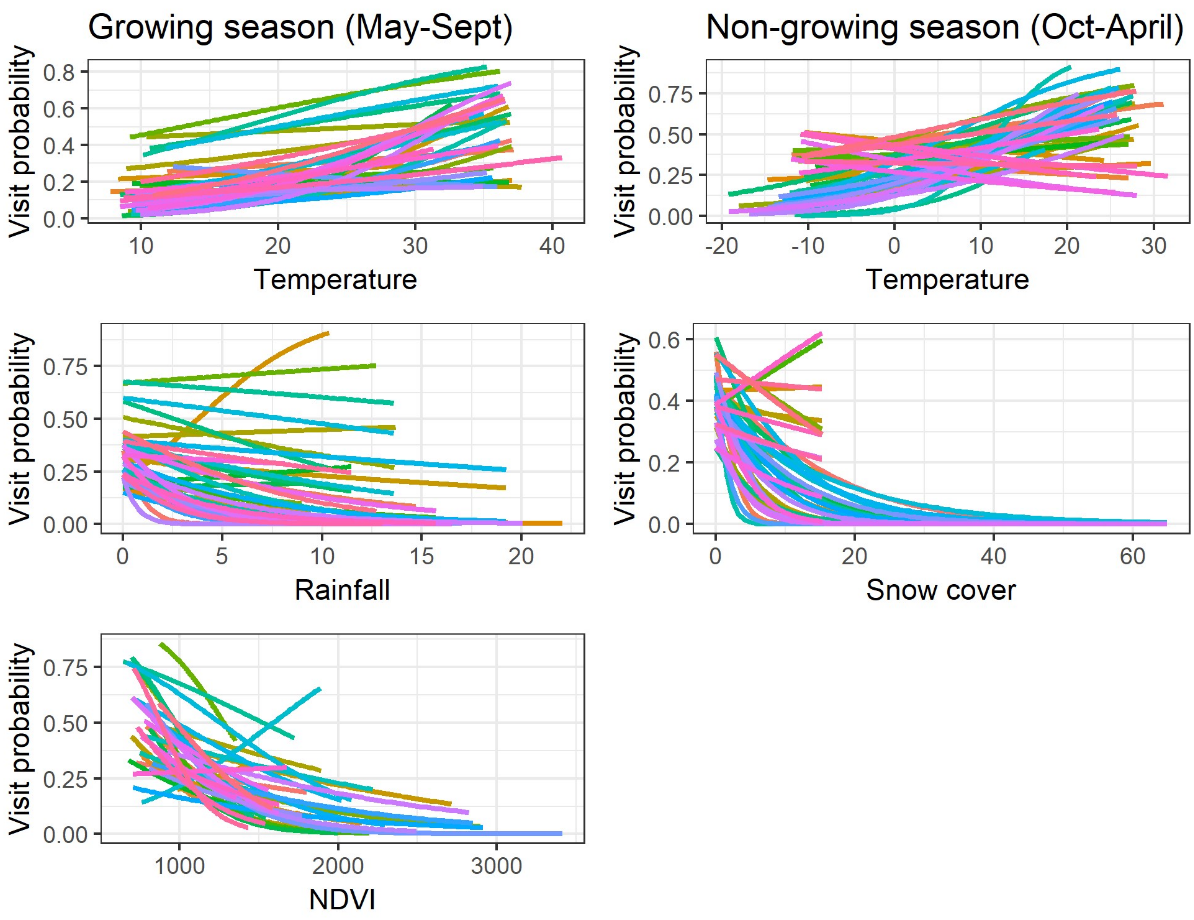

Fig. S4: Individual variation in the probability of individual khulan visiting a waterpoint as a function of climate variables and NDVI in the Southern Gobi Region, August 2013 to March 2018. Each line represents a different animal.

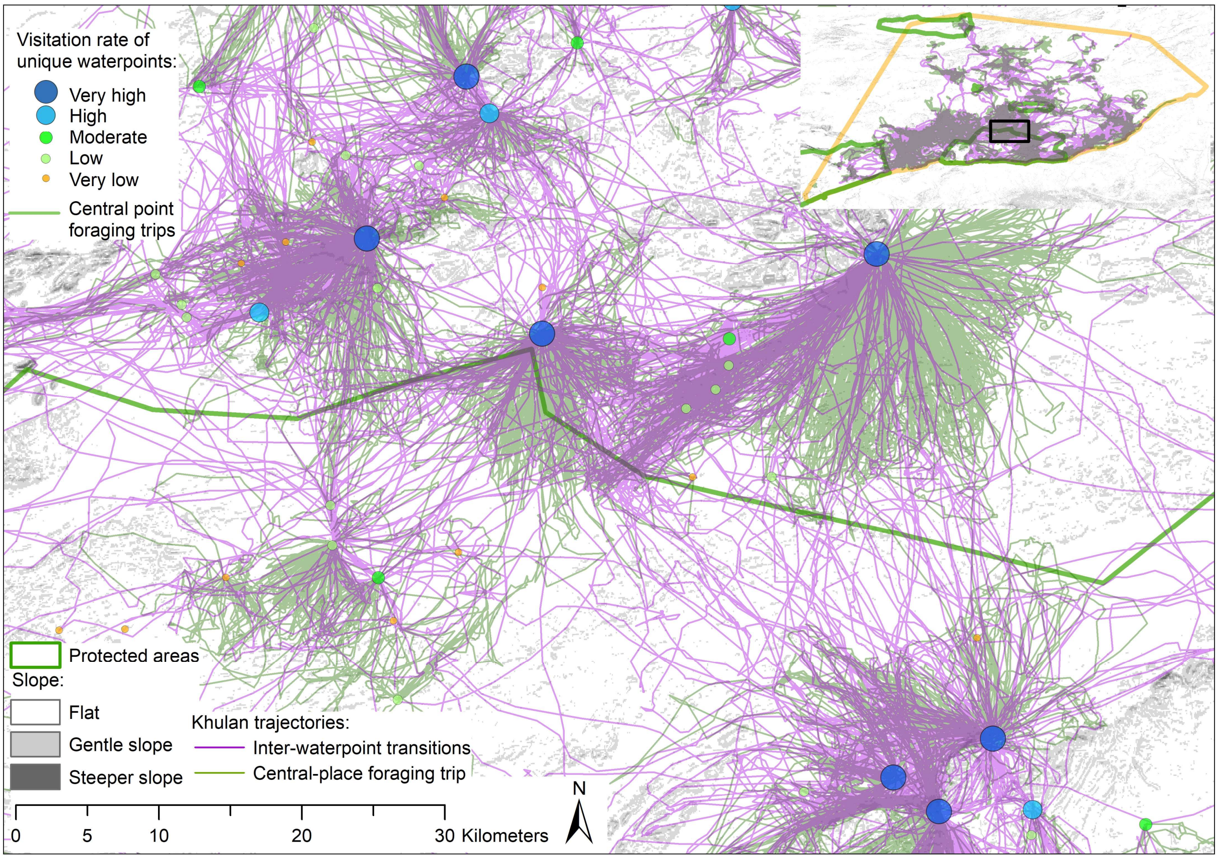

Fig. S5: Zoomed-in view of khulan trajectories converging at waterpoints in the South Gobi Region.

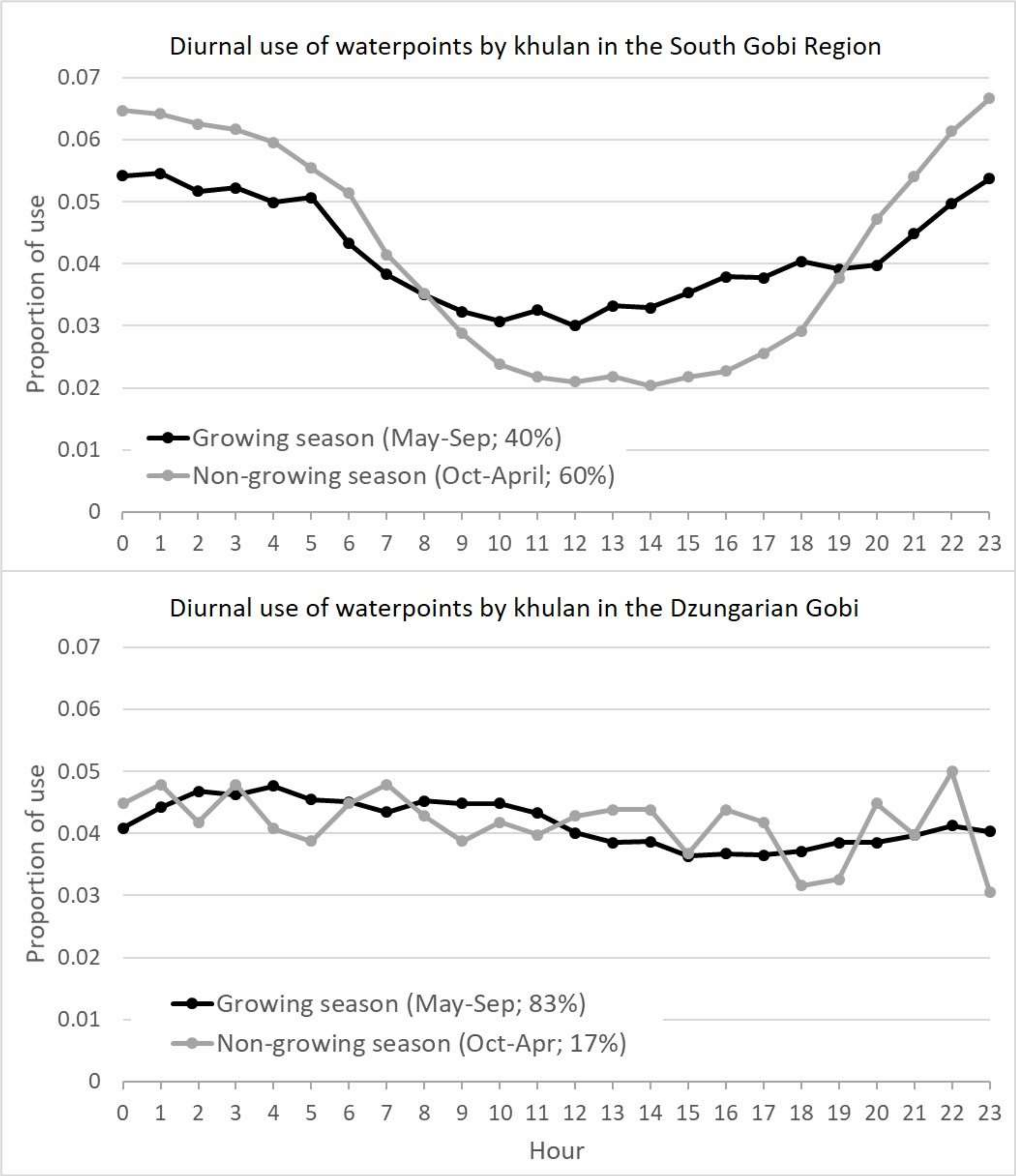

Fig. S6: Diurnal use of waterpoints in the South Gobi Region as compared to the Dzungarian Gobi in south-western Mongolia (Source: PK, JP unpubl. data from 7 khulan monitored with GPS collars for 12 months in 2007/8 or 2009/10).

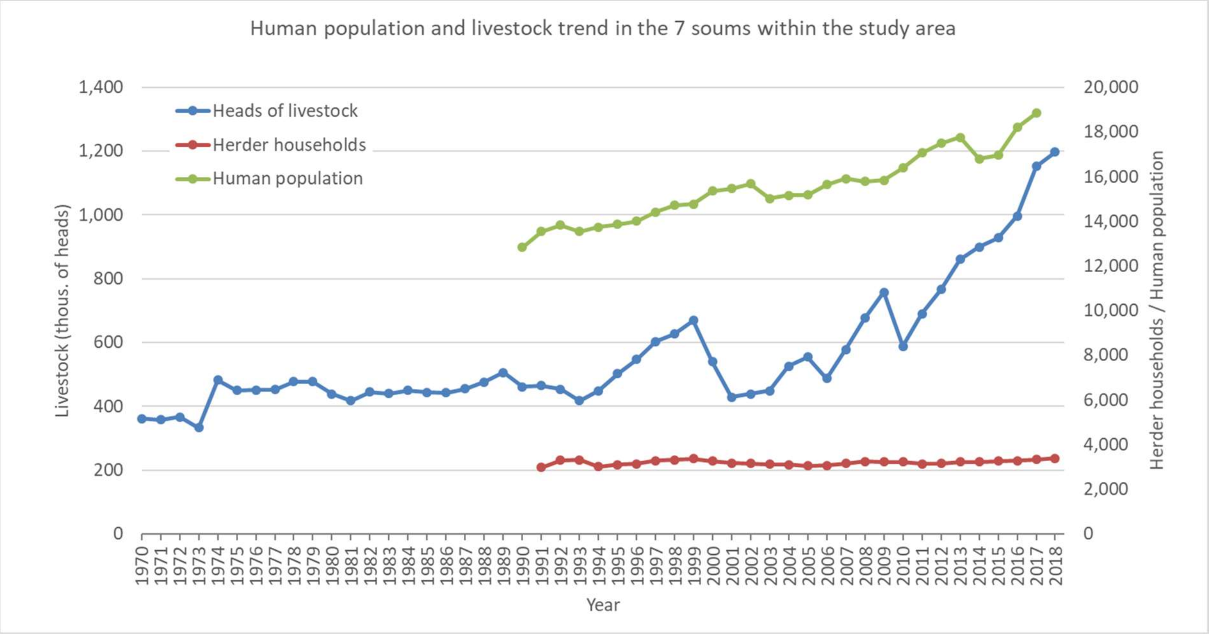

Fig. S7: Trends in the number of livestock (total sheep, goats, horses, camels, and cattle), herder households, and human population within the 7 soums Bayan-Ovoo, Khanbogd, Khatanbulag, Khovsgol, Ulaanbadrakh, Manlai, Mandakh which intersect the study area in the South Gobi Region. The increase in the total population since 2005 is almost entirely due to population increase in Khanbogd soum, where the Oyu Tolgoi mine is located. Source: Mongolian Statistical Information Service 2019 at http://www.1212.mn/en/ (data downloaded on 16.02.2019).

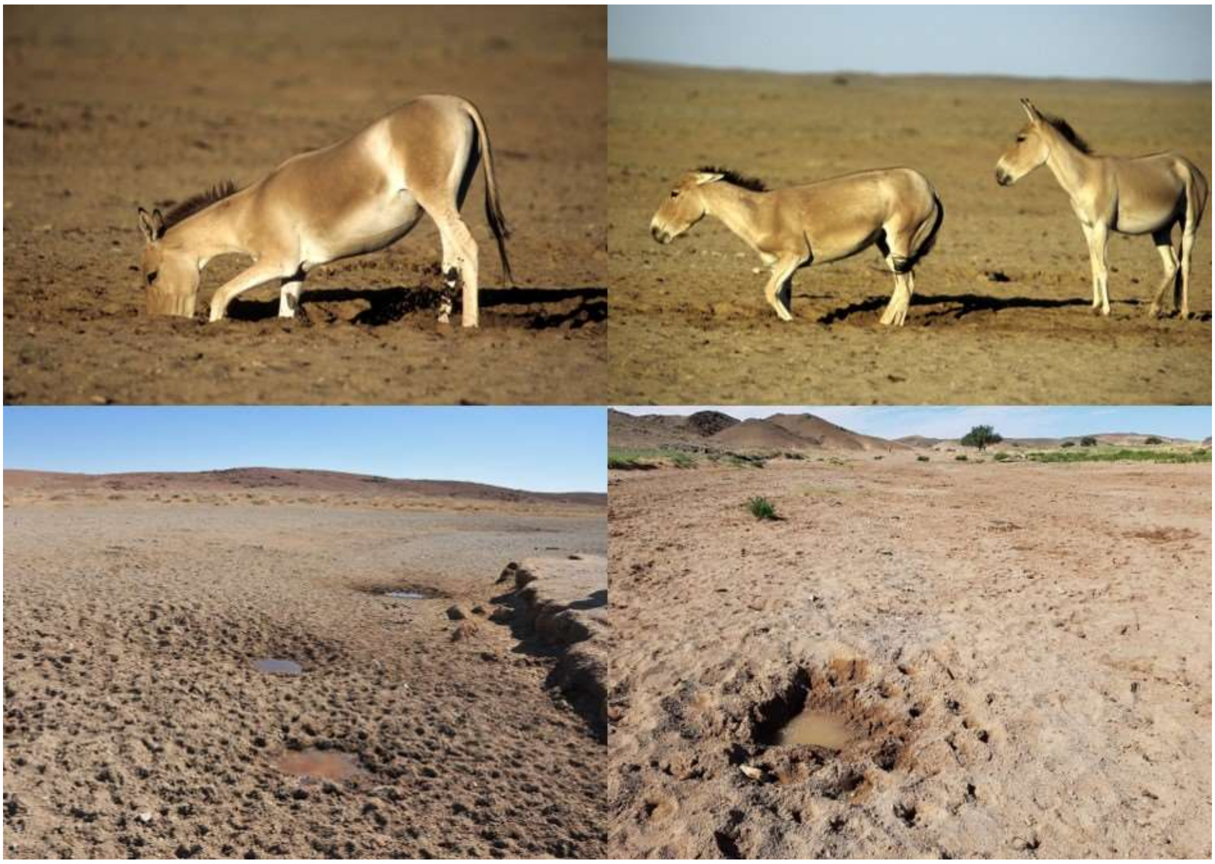

Fig. S8: Khulan diggings in dry riverbeds in the South Gobi Region. *Photos: P. Kaczensky*

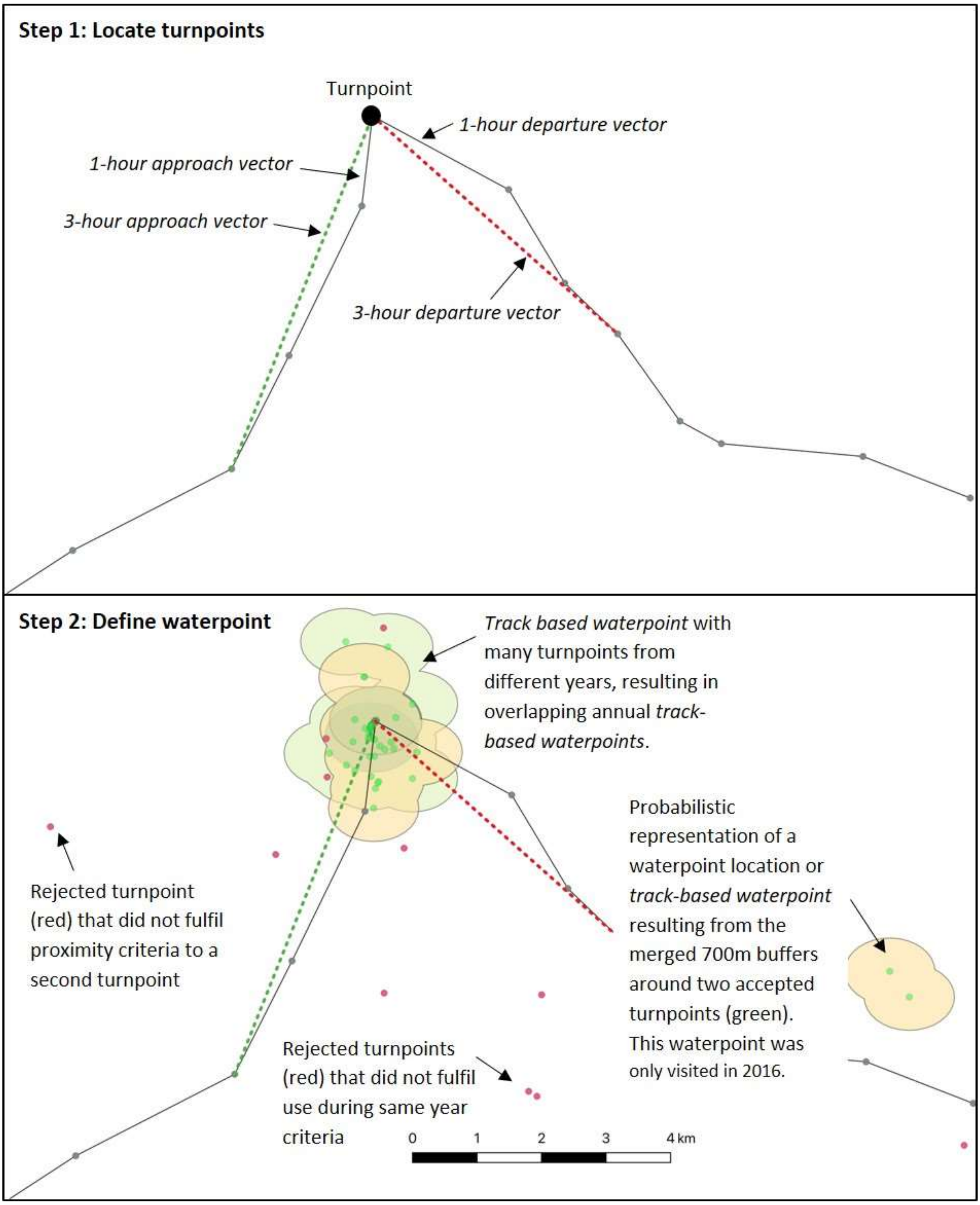

Fig. S9: Two step water-finding algorithm. A) Step 1: Turnpoints were identified based on turn angles of the 3-hour and 1-hour approach and departure vectors ($\alpha = \beta \leq 90^o$) and the length of the 3-hour approach and departure vectors (L ≥ 2 km) along each khulan track. B) Step 2: Annual waterpoints were identified based on the number of other turn points from the same year (t ≥ 2) within a buffer with 700m diameter around each turnpoint. Those turnpoints failing to meet the criteria were dropped, the overlapping buffers of the remaining turnpoints were used to delineate annual waterpoints. Those with different colored buffers are from different years and if overlapping identify waterpoints utilized by khulan in multiple years.

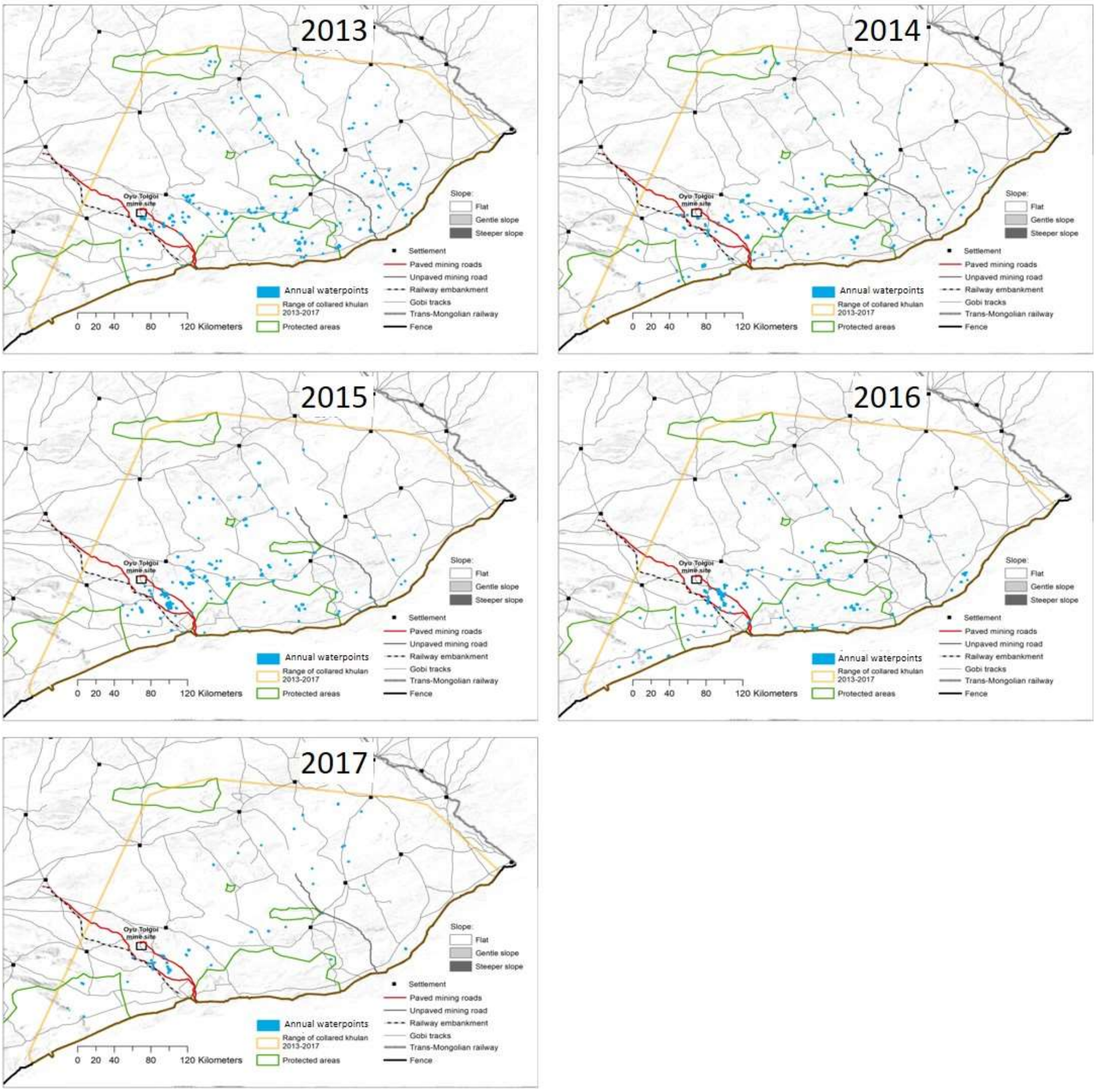

Fig. S10: Shape and size of the annual waterpoints in the South Gobi Region. The size of the waterpoint shapes were enlarged for better visibility. Year-long periods were defined from August 1 – July 31. The final period (2017) was truncated, and ended on 31 March 2018.

Table S1: Khulan collared and monitored from August 2013 to March 2018 in the South Gobi Region. Age is based on tooth eruption pattern up to 5 years and overall tooth wear thereafter; Total range is the size of the 100% Minimum Convex Polygon (MCP) around all GPS locations over the entire monitoring time of each khulan (in the south the area was clipped by the fenced international border).

Deployment year	Collar ID	Sex	Age (based on tooth wear)	Capture location		Start date (M/D/Y)	End date (M/D/Y)	Days tracked	Total range (km^2)
				Longitude	Latitude				
2013	13549	female	10	107.14362	42.77890	8/25/13	8/20/15	725	24,260
2013	13555	female	10	109.22859	43.39125	8/28/13	5/16/15	626	45,143
2013	13556	male	4	109.33645	43.41272	8/29/13	5/26/14	270	17,910
2013	13557	female	4	109.09394	43.29222	8/30/13	8/20/15	720	44,690
2013	13741	male	10.5	109.27354	43.45523	8/29/13	8/20/15	721	25,732
2013	13742	male	5	107.12234	42.80311	8/25/13	1/14/15	507	51,535
2013	13743	male	11	109.19852	43.46243	8/28/13	8/20/15	722	10,971
2013	13744	female	2	109.18433	43.44719	8/29/13	8/20/15	721	43,735
2013	13745	female	9	106.86977	42.90927	8/24/13	8/21/15	727	44,952
2013	13746	male	2	106.87460	42.91884	8/24/13	5/28/14	277	48,125
2013	13747	male	7	109.20031	43.27670	8/28/13	12/3/15	827	42,052
2013	13748	male	3	106.83424	42.95559	8/24/13	7/27/15	702	22,258
2013	34406	male	3	106.97776	42.84034	8/25/13	6/10/15	654	63,606
2013	34407	male	10	106.98048	42.83515	8/26/13	8/20/15	724	36,846
2013	34408	female	6.5	106.78283	42.93926	8/24/13	3/11/15	564	20,391
2013	34409	male	2	107.05373	42.76232	8/23/13	7/4/15	680	56,975
2013	34410	male	13	109.21400	43.43226	8/29/13	6/6/14	281	13,834
2013	34411	female	7	109.17558	43.34399	8/30/13	7/31/15	700	54,383
2013	34412	male	9	106.92326	42.85182	8/26/13	7/5/15	678	28,272
2013	34413	male	10	108.68736	43.29558	8/28/13	8/20/15	722	15,632
2015	13155_3	female	6.5	106.93651	42.55289	10/16/15	10/16/16	366	22,389
2015	13555_2	male	10	108.90009	43.26763	10/19/15	10/7/17	719	25,554

2015	13556_2	female	12	108.95409	43.27906	10/19/15	10/10/17	722	31,062
2015	13742_2	female	10	109.07966	43.23849	10/19/15	9/5/17	687	46,696
2015	19835	male	15	107.22105	42.73127	10/15/15	8/25/17	680	11,197
2015	19836	female	15	108.91349	43.30989	10/19/15	1/25/16	98	19,449
2015	19837	female	5	107.08775	42.77804	10/15/15	8/23/17	678	31,377
2015	19838	male	8	108.95925	43.27881	10/19/15	4/14/16	178	17,407
2015	19839	female	5.5	107.06463	42.56510	10/17/15	12/6/17	781	23,678
2015	19840	male	7	107.01736	42.53722	10/16/15	10/17/16	367	31,804
2015	19841	female	4.5	109.02758	43.30498	10/18/15	12/10/15	53	15,345
2015	19842	male	8	107.28164	42.73559	10/15/15	12/31/17	808	25,898
2015	19843	female	6	107.10968	42.56804	10/16/15	12/31/17	807	37,543
2015	19844	male	10	109.09396	43.29465	10/19/15	10/14/17	726	32,153
2015	19845	female	10	107.19285	42.71736	10/15/15	12/31/17	808	50,562
2015	19847	female	6	107.15724	42.78597	10/15/15	12/31/17	808	57,040
2015	19848	female	12	106.98009	42.54682	10/16/15	11/22/17	768	56,798
2015	19849	female	5	107.16116	42.74303	10/17/15	10/29/16	378	38,133
2015	19850	male	7	109.06090	43.28752	10/19/15	4/29/16	193	19,891
2015	19851	female	10	109.05108	43.32692	10/19/15	5/5/17	564	35,538
2015	19852	male	10	109.00198	43.25988	10/19/15	12/31/17	804	36,921

R script 1: Hierarchical cluster analysis to categorize waterpoint.

```
#########################################################################################
# Hierarchical cluster analysis to categorize waterpoint use into groups based on the number of total
#visits, the number of unique individuals of the visits, and the number of years the waterpoint was
#visited
#########################################################################################
# Ward Hierarchical Clustering
mydata <- wpDF[,c("nvisits","n_collars","n_periods")]
scale <- function(x) (x-min(x))/(max(x)-min(x))
mydata$nvisits <- scale(mydata$nvisits)
mydata$n_collars <- scale(mydata$n_collars)
mydata$n_periods <- scale(mydata$n_periods)
d <- dist(mydata, method = "euclidean") # distance matrix
fit <- hclust(d, method="ward")
plot(fit)

#########################################################################################
#deciding number of groups
mydata<-mydata[,1:3]
wss <- (nrow(mydata)-1)*sum(apply(mydata,2,var))
for (i in 2:15) wss[i] <- sum(kmeans(mydata,
                                   centers=i)$withinss)
qplot(1:15, wss, geom=c("point","line"), xlab="Number of Clusters",
     ylab="Within groups sum of squares")+theme_bw()
ggsave(filename="Cluster_number_choice.tiff",width=4,height=3)

#5 seems the elbow
groups <- cutree(fit, k=5)
wpDF$group<-groups
table(wpDF$group)
length(wpDF$permanent_)

#########################################################################################
#plot the clusters
#relabel group levels
wpDF$group <- as.factor(wpDF$group)
levels(wpDF$group) <-
  c("Moderate",
    "High",
    "Very low",
    "Very high",
    "Low")

#reorder the levels
wpDF$Priority <- factor(wpDF$group, levels=rev(c("Very low",
                                      "Low",
                                      "Moderate",
                                      "High",
                                      "Very high")))

q1 <- qplot(nvisits,n_collars,data=wpDF,colour=Priority)+xlab("N Visits") + ylab("N Indivs")+
```

```
  scale_colour_manual(values=rev(c("orange","darkolivegreen2","green",
                              "cyan4","darkblue")))
q2 <- qplot(nvisits,n_periods,data=wpDF,colour=Priority)+xlab("N Visits") + ylab("N Years")+
  scale_colour_manual(values=rev(c("orange","darkolivegreen2","green",
                              "cyan4","darkblue")))
library(cowplot)
plot_grid(q1,q2,ncol=1)
ggsave(filename="Use_classes.tiff",width=4.5,height=4)

#create dataframe
mydata <- wpDF[,c("nvisits","n_collars","n_periods")]
mydata$group<-wpDF$group
mydata$waterpointID<-wpDF$permanent_
write.csv(mydata,file="waterpoint_by_use_group_6.csv",row.names=FALSE)

#plot by number of visits
library(ggplot2)

#coords.x1,y=coords.x2
wpDF$coords.x1 <- wpDF$centroid_longitude
wpDF$coords.x2 <- wpDF$centroid_latitude

#plot total number of visits
hist(wpDF$nvisits)
wpDF$log.nuVisits <- log(wpDF$nvisits)
wpDF <- wpDF[order(wpDF$log.nuVisits,decreasing = F),]
g1 <- ggplot(data=wpDF,aes(x=coords.x1,y=coords.x2))+
  geom_point(aes(colour=log.nuVisits),
           size=rel(0.75))+
  scale_colour_gradient2(low="orange",mid="green",high="darkblue",
                        midpoint=3)+
  theme_minimal()+xlab("Longitude")+ylab("Latitude")+
  labs(color = "log N Visits\n")

#plot by total number of individuals
hist(wpDF$n_collars)
wpDF$log.nuIndivs <- log(wpDF$n_collars)
wpDF <- wpDF[order(wpDF$log.nuIndivs,decreasing = F),]
g2 <- ggplot(data=wpDF,aes(x=coords.x1,y=coords.x2))+
  geom_point(aes(colour=log.nuIndivs),size=rel(0.75))+
  scale_colour_gradient2(low="orange",mid="green",high="darkblue",
                        midpoint=1.5)+
  theme_minimal()+xlab("Longitude")+ylab("Latitude")+
  labs(color = "log N Indivs\n")

#plot by total number of years
hist(wpDF$n_periods)
wpDF$nuYears <- wpDF$n_periods
wpDF <- wpDF[order(wpDF$nuYears,decreasing = F),]
g3 <- ggplot(data=wpDF,aes(x=coords.x1,y=coords.x2))+
  geom_point(aes(colour=nuYears),size=rel(0.75))+
  scale_colour_gradient2(low="orange",mid="green",high="darkblue",limits=c(1,5),midpoint=3)+
```

```
  theme_minimal()+xlab("Longitude")+ylab("Latitude")+
  labs(color = "N Year   \n")

#combine all plots
library(cowplot)
plot_grid(g1,g2,g3)
###########################################################################################
```

R-script 2: GAM to test for variables influencing khulan visiting waterpoints.

```
#######################################################################################
#GAM – Variables influencing probability of khulan visiting waterpoints
#######################################################################################
#read in individuals level data and format it
df<- read.csv("visits_by_date_and_collar_31mar2018.csv")
head(df)

#format date and extract year and month info
library(lubridate)
df$date_local<-as.Date(df$date_local)
df$Month<-month(df$date_local)


#for simplicity, convert n visits as a binary, yes/no, variable
df$n_visitB<-ifelse(df$n_visits>0,1,0)
table(df$n_visitB)

#######################################################################################
#changing projection of coordinates into metres

library(sp)
equalareaProj<-"+proj=utm +zone=48 +datum=WGS84 +units=m +no_defs "
coords<-df[,c("longitude","latitude")]
coordinates(coords)<-c("longitude","latitude")
proj4string(coords)<-CRS("+proj=longlat +datum=WGS84")
coords<-spTransform(coords,CRS(equalareaProj))
df$x <- coords@coords[,1]
df$y <- coords@coords[,2]

#######################################################################################

#plotting individual random effects
#plotting indiv random effects
library(ggplot2)

#summer
q1<-ggplot(subset(df,Month %in% 5:9))+
  stat_smooth(aes(x=avgtemp_day,y=n_visitB,colour=factor(id_collar)),
        method="glm",method.args = list(family = binomial),se=F)+
  theme_bw()+xlab("Temperature")+ylab("Visit probability")+
  theme(legend.position = "none")+ggtitle("Growing season (May-Sept)")

q2<-ggplot(subset(df,Month %in% 5:9))+
  stat_smooth(aes(x=gpcc_rain_day,y=n_visitB,colour=factor(id_collar)),
        method="glm",method.args = list(family = binomial),se=F)+
  theme_bw()+xlab("Rainfall")+ylab("Visit probability")+
  theme(legend.position = "none")

q3<-ggplot(subset(df,Month %in% 5:9))+
  stat_smooth(aes(x=ndvi_avg_day,y=n_visitB,colour=factor(id_collar)),
```

```
            method="glm",method.args = list(family = binomial),se=F)+
  theme_bw()+xlab("NDVI")+ylab("Visit probability")+
  theme(legend.position = "none")

#winter
q5<-ggplot(subset(df,!Month %in% 5:9))+
  stat_smooth(aes(x=avgtemp_day,y=n_visitB,colour=factor(id_collar)),
            method="glm",method.args = list(family = binomial),se=F)+
  theme_bw()+xlab("Temperature")+ylab("Visit probability")+
  theme(legend.position = "none")+ggtitle("Non-growing season (Oct-April)")

q6<-ggplot(subset(df,!Month %in% 5:9))+
  stat_smooth(aes(x=snowavg_day,y=n_visitB,colour=factor(id_collar)),
            method="glm",method.args = list(family = binomial),se=F)+
  theme_bw()+xlab("Snow cover")+ylab("Visit probability")+
  theme(legend.position = "none")

library(cowplot)
plot_grid(q1,q5,
        q2,
        q6,q3,ncol=2)

##########################################################################################
#analysis by season, including lat and lon

library(mgcv)
library(boot)
library(ggplot2)
library(gamm4)

########
#winter#
#######
dfW <- subset(df,!Month %in% 5:9)

#remove rows with missing values
dfW <- subset(dfW, !is.na(snowavg_day))
dfW <- subset(dfW, !is.na(avgtemp_day))

#standardize covarites
dfW$s_snowavg_day <- scale(dfW$snowavg_day)
dfW$s_avgtemp_day <- scale(dfW$avgtemp_day)
dfW$s_median_maxtemp_day <- scale(dfW$median_maxtemp_day)

summary(dfW)

#run gamm
gamW <- gamm4(n_visitB ~ s_snowavg_day +
              s_avgtemp_day +
              s(longitude,latitude,bs="ds"),
            random = ~ (1|id_collar) +
              (0+s_snowavg_day|id_collar) +
```

```
            (0+s_avgtemp_day|id_collar),
           data=dfW,family=binomial)
summary(gamW$mer)
summary(gamW)
#AIC      BIC   logLik deviance df.resid
#16446.9  16515.6  -8214.5  16428.9    15162

########
#summer#
#######
dfS <- subset(df, Month %in% 5:9)

#remove rows with missing data
dfS <- subset(dfS, !is.na(avgtemp_day))

#standardize covariates
dfS$s_avgtemp_day <- scale(dfS$avgtemp_day)
dfS$s_median_maxtemp_day <- scale(dfS$median_maxtemp_day)
dfS$s_gpcc_rain_day <- scale(dfS$gpcc_rain_day)
dfS$s_ndvi_avg_day <- scale(dfS$ndvi_avg_day)
summary(dfS)

#fit gamm
gamS <- gamm4(n_visitB ~ s_avgtemp_day +
            s_gpcc_rain_day +
            s_ndvi_avg_day +
            s(longitude,latitude,bs="ds"),
           random = ~ (1|id_collar) +
            (0+s_avgtemp_day|id_collar) +
            (0+s_gpcc_rain_day|id_collar) +
            (0+s_ndvi_avg_day|id_collar),
           data=dfS,family=binomial)
summary(gamS$mer)
summary(gamS)
#AIC      BIC   logLik deviance df.resid
#9770.2   9848.5  -4874.1   9748.2     9118

##################################################################################
#get explanatory power
summary(gamW$gam)#R-sq.(adj) =  0.186
summary(gamS$gam)#R-sq.(adj) =  0.146

##################################################################################
#plotting:
summerCoefs<-data.frame(Estimate=as.numeric(summary(gamS$gam)$p.coeff))
summerCoefs$Param<-names(summary(gamS$gam)$p.coeff)
confintCoefs<-data.frame(confint(gamS$gam)[1:4,])
summerCoefs<-cbind(summerCoefs,confintCoefs)
summerCoefs$Season<-"Growing season (May-September)"

winterCoefs<-data.frame(Estimate=as.numeric(summary(gamW$gam)$p.coeff))
winterCoefs$Param<-names(summary(gamW$gam)$p.coeff)
```

```
confintCoefs<-data.frame(confint(gamW$gam)[1:3,])
winterCoefs<-cbind(winterCoefs,confintCoefs)
winterCoefs$Season<-"Non-growing season (October-April)"

#relabel the variable:
allCoefs<-rbind(summerCoefs,winterCoefs)
allCoefs$Param[which(allCoefs$Param=="s_snowavg_day")]<-"Snow cover"
allCoefs$Param[which(allCoefs$Param=="s_avgtemp_day")]<-"Temperature"
allCoefs$Param[which(allCoefs$Param=="s_ndvi_avg_day")]<-"NDVI"
allCoefs$Param[which(allCoefs$Param=="s_gpcc_rain_day")]<-"Rainfall"
allCoefs$Param <- as.factor(allCoefs$Param)
allCoefs$Param <- factor(allCoefs$Param, levels=rev(c("(Intercept)","Temperature","NDVI",
                                           "Rainfall","Snow cover")))

#plotting coefficients
ggplot(subset(allCoefs,Param!="(Intercept)"))+
  geom_crossbar(aes(x=Param,y=Estimate,ymin=X2.5..,ymax=X97.5..))+
  geom_hline(yintercept=0,color="black",linetype="dashed")+
  facet_wrap(~Season,ncol=2)+
  coord_flip()+
  theme_bw()+
  ylab("Effect on waterpoint-use")+xlab("")
##########################################################################################
```
